# Acquired Hemophilia as a Paraneoplastic Syndrome in a Patient With Small Cell Lung Carcinoma

**DOI:** 10.7759/cureus.23926

**Published:** 2022-04-07

**Authors:** James F Lyon, Alina Basnet

**Affiliations:** 1 Hematology Oncology, State University of New York (SUNY) Upstate Medical University, Syracuse, USA

**Keywords:** immunotherapy adverse effect, factor viii inhibitor, acquired hemophilia, small cell lung carcinoma, paraneoplastic syndrome

## Abstract

This case report describes a 72-year-old female patient diagnosed with small cell lung carcinoma who was found to have elevated partial thromboplastin time (PTT) after reporting diarrhea and melanotic stool. Further investigations revealed the presence of a factor VIII inhibitor, possibly resulting from a side effect of immunotherapy or of possible paraneoplastic origin. The patient’s PTT remained elevated following a course of steroid treatment, raising the likelihood of paraneoplastic etiology. This case represents a rare paraneoplastic syndrome, with a previously unreported presentation of melanotic stool as opposed to an acute bleeding episode.

## Introduction

Small cell lung carcinoma (SCL) is an aggressive pulmonary malignancy associated with early locoregional and distant metastasis, representing approximately 13% of all newly diagnosed lung cancers [1,2]. It is associated with several well-established paraneoplastic syndromes, including ectopic Cushing syndrome, hyponatremia of malignancy and Lambert-Eaton myasthenic syndrome [3]. These paraneoplastic syndromes fall into two broad classes: those mediated by ectopic production of biologically active peptides and those caused by antibody or cell-mediated immune responses.

Acquired hemophilia, meanwhile, is the most common acquired disease affecting clotting factors [4]. Possible causes that have been empirically described include autoimmune disease (such as rheumatoid arthritis or systemic lupus erythematosus), malignancy (most frequently solid tumors such as SCL), dermatologic disorders (such as psoriasis or pemphigus), postpartum changes or drug interactions [5,6]. Acquired hemophilia occurs due to either acquired deficiency of factor VIII or development of a factor VIII inhibitor in the body. Here, we describe a rare case of acquired hemophilia in a 72-year-old female diagnosed with SCL, who presented with melanotic stool and elevated partial thromboplastin time (PTT). Further studies revealed the presence of a factor VIII inhibitor with possible paraneoplastic origin.

## Case presentation

**Figure 1:**
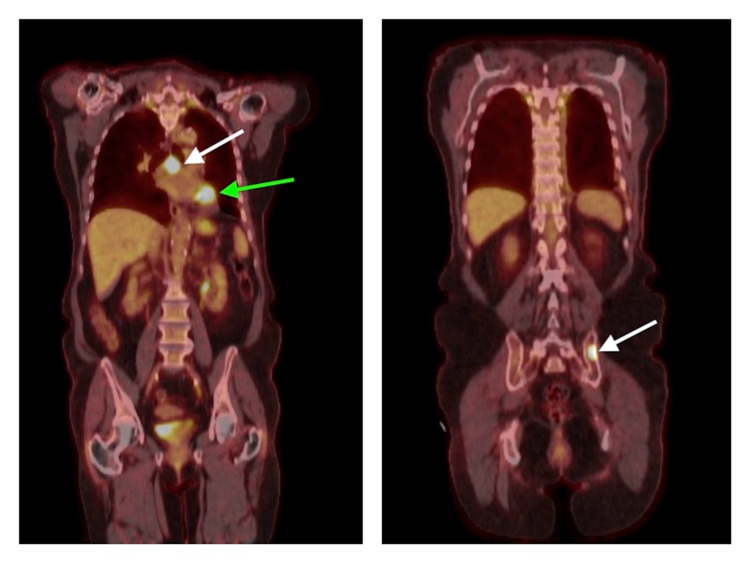
PET-CT scan from March 2020 Left: PET-CT scan from March 2020 following left lower lobectomy, demonstrating focal activity and soft tissue nodularity in the left hilar surgical site (indicated by green arrow) as well as metabolically active subcarinal lymph nodes (indicated by white arrow), concerning for metastatic disease. Right: PET-CT scan from March 2020, which revealed focal activity in the left ilium (as indicated by the white arrow), concerning for osseous metastatic disease. PET-CT: positron emission tomography-computed tomography.

The patient is a 72-year-old female, with a past medical history of chronic kidney disease, gastroesophageal reflux disease, peripheral artery disease, hypertension and hypercholesterolemia, who presented as a new consult after diagnosis of atypical carcinoid tumor of the left lower lobe of the lung in November 2019. This diagnosis was made on the basis of histopathological analysis following biopsy. A left lower lobectomy was performed in January 2020, with pathology revealing a prominent small cell component, and mediastinal lymph node sampling revealing metastasis to the peribronchial lymph nodes as well as a level 7 (subcarinal) lymph node. Staging scans, including an MRI of the brain and a positron emission tomography-computed tomography (PET-CT) scan, revealed evidence of bony metastasis in the left ilium (Figure 1).

**Figure 2:**
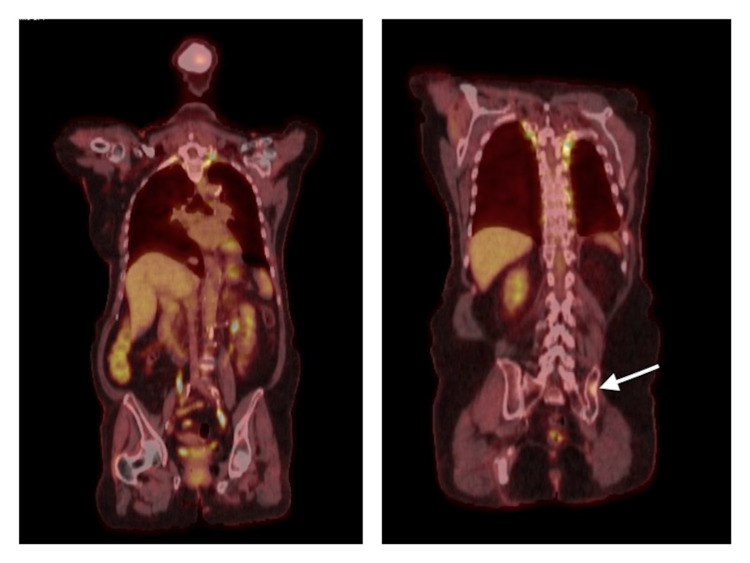
PET-CT scan from June 2020 Left: PET-CT scan from June 2020 following initiation of systemic chemotherapy, revealing resolution of left hilar and subcarinal FDG-avid lesions without new hypermetabolic disease. Right: PET-CT scan from June 2020 following initiation of systemic chemotherapy, demonstrating significantly decreased FDG activity within previous left iliac sclerotic lesion (as indicated by white arrow). PET-CT: positron emission tomography-computed tomography, FDG: fluorodeoxyglucose.

The patient was deemed an appropriate candidate for systemic treatment. Organ profiles prior to the initiation of the treatment showed decreased glomerular filtration rate (GFR) (of 26 mL/min) with normal cardiac and hepatic function testing. She was initiated on carboplatin-etoposide-atezolizumab with dose reductions, given her advanced age and frail performance status. Further scans four months later showed a favorable response to treatment (Figure 2), and the patient was initiated on maintenance immunotherapy with atezolizumab.

Two months after the initiation of atezolizumab, the patient reported experiencing diarrhea and melanotic stool, without other episodes of bleeding, such as epistaxis, ecchymosis or hematemesis. At this time, immunotherapy was held and an increased dose of pantoprazole was prescribed for medical management. Due to gastrointestinal bleeding, a coagulation workup was done, revealing an elevated PTT of 72.9 seconds (normal range: 24.0 to 33.0 seconds), which prior to her diagnosis of SCL had been only mildly elevated to 36.0 seconds. Other lab studies performed at this time included a complete blood count, revealing a hemoglobin of 8.4 g/dL and a platelet count of 274 platelets/mL, as well as a complete metabolic panel, revealing normal liver enzymes. A subsequent mixing study did not normalize the PTT, suggesting the presence of an inhibitor. The titer of the inhibitor was elevated at 5.8 Bethesda units (normal range: less than 0.6 Bethesda units). The possible etiologies considered for the appearance of a factor VIII inhibitor included an adverse effect of atezolizumab therapy versus a possible paraneoplastic syndrome associated with her SCL. The patient was initiated on corticosteroids due to suspicion of possible immunotherapy-related colitis, leading to improvement in her diarrhea. The patient’s PTT, however, remains elevated. The acquired factor VIII inhibitor was considered to be more likely of paraneoplastic origin, based on the reasoning that the patient's PTT remained elevated despite cessation of the immunotherapy and treatment with corticosteroids. The patient received consolidative thoracic radiation therapy the following month and was continued on maintenance atezolizumab. The patient elected to discontinue her immunotherapy, due to the adverse effect of diarrhea, and has since remained under surveillance. Recent scans have shown stable disease without evidence of progression.

## Discussion

This report presents a unique case of acquired hemophilia in a patient diagnosed with SCL, who developed a factor VIII inhibitor as a paraneoplastic syndrome. While SCL is associated with a number of well-established paraneoplastic syndromes, acquired hemophilia has been rarely described in the literature [7,8]. It is possible that this patient’s acquired hemophilia represents an adverse effect associated with the patient’s use of immunotherapy, namely atezolizumab. Atezolizumab is an immune checkpoint inhibitor that has been associated with a variety of immune-related adverse reactions, most frequently affecting the liver, lungs, pituitary, thyroid and skin [9]. The development of acquired hemophilia in patients on immune checkpoint inhibitors has been described [10,11]. In these reported cases, following hemodynamic stabilization, the elevation of PTT resolved with immunosuppressive treatment. However, while the patient’s diarrhea improved with steroids, the failure of PTT to normalize with steroids suggests another etiology.

It is estimated that up to 8% of cancer patients will be affected by a paraneoplastic syndrome [12]; however, as the number of cancer patients grows and as they are able to live longer with the disease, this number is expected to rise. These syndromes are increasingly well-characterized with effective treatment options, allowing for improvement in quality of life for these patients as well as prolonged survival and improved delivery of cancer therapy. Resolution of paraneoplastic syndromes, including acquired hemophilia, may occur with successful treatment of the underlying malignancy. Depending on the specific paraneoplastic syndrome with which a patient presents, immunosuppression, such as with corticosteroids or rituximab, and correction of specific electrolyte or hormonal derangements may also be effective treatment options [13].

This case presentation differs from those previously described in the literature in several ways [7,8]. In other cases, patients experienced acute hemorrhagic bleeding episodes, while in this case report, the patient reported only melanotic stool [7,8]. Additionally, in one previous report, the patient's bleeding episode and abnormal coagulation studies resolved with high-dose steroids, and in another, the patient's levels of factor VIII inhibitor became undetectable following a favorable response to chemotherapy. However, in this case, the patient's PTT remains elevated, despite stable disease.

## Conclusions

Small cell lung carcinoma is an aggressive pulmonary malignancy associated with several well-established paraneoplastic syndromes. In this case report, we present a unique case of acquired hemophilia in a patient with small cell carcinoma without an acute bleeding episode. Resolution of paraneoplastic syndromes may occur with successful treatment of the underlying malignancy or alternatively with treatment using corticosteroids.
